# Effects of high-intensity interval training on executive functions and IGF-1 levels in sedentary young women: a randomized controlled trial

**DOI:** 10.3389/fspor.2025.1597171

**Published:** 2025-06-04

**Authors:** Manuel Jesús Jimenez-Roldán, Borja Sañudo Corrales, Luis Carrasco Páez

**Affiliations:** ^1^ Department of Physical Education and Sport, University of Osuna, Seville, Spain; ^2^ Department of Physical Education and Sport, University of Seville, Seville, Spain

**Keywords:** executive functions, HIIT, IGF-1, women, sedentary behavior

## Abstract

**Purpose:**

Sedentary behavior and physical inactivity are widespread among university students, negatively impacting physical and neurocognitive health. Executive functions and insulin-like growth factor 1 (IGF-1) are adversely affected by inactivity. Moreover, physical activity increases IGF-1 concentrations, which may mediate beneficial effects on brain health and cognitive performance. The effect of HIIT exercise combined with increased physical activity on these variables is not well understood. This study evaluated the chronic effects of HIIT, with or without increased physical activity, over 12 weeks of training and a 12-week follow-up period, on executive functions and IGF-1 concentrations in sedentary young university students.

**Method:**

This randomized controlled trial included 77 sedentary female university students, assigned to three groups: HIIT (*n* = 25), HIIT plus increased daily physical activity (HIIT + PA, *n* = 25), and control group (CG, *n* = 27). The intervention involved assessments at baseline (T1), post-intervention (T2), and after a 12-week follow-up (T3). Salivary IGF-1 concentrations were measured using enzyme-linked immunosorbent assay (ELISA). Executive functions were evaluated with the Stroop Test, Wisconsin Card Sorting Test, and Digit Span Test. The HIIT protocol consisted of 40-min sessions, three times per week, delivered online. The HIIT + PA group also aimed to complete 10,000 steps per day.

**Results:**

No statistically significant changes in IGF-1 concentrations were found over time or between groups, although descriptive increases were observed in both HIIT and HIIT + PA groups at follow-up. For executive functions, improvements over time were found in cognitive flexibility (WCST) and working memory (DST-B and DST-T), but without significant group × time interactions. Only WCST-E-P showed a significant group effect (*p* = 0.028), indicating possible differences between groups in cognitive rigidity. The HIIT + PA group showed a tendency toward improved inhibitory control (Stroop accuracy), although this was not supported by significant interaction effects. Working memory improvements (DST-B) were significant over time, especially in the HIIT group, but again without significant differences between groups.

**Conclusion:**

A 12-week HIIT program, with or without increased daily physical activity, can lead to improvements in executive functions in sedentary young women, particularly in working memory and cognitive flexibility. However, these changes were not exclusive to the intervention groups, suggesting possible contributions from repeated testing or other external factors. While IGF-1 levels showed upward trends, no significant group-level effects were confirmed. These findings highlight the potential cognitive benefits of HIIT but emphasize the need for further research with tighter controls and objective neurobiological measures to clarify the mechanisms involved. Incorporating HIIT into student routines may support cognitive health, but broader lifestyle interventions may be needed to sustain long-term benefits.

## Introduction

Sedentary behavior and physical inactivity are prevalent issues among young populations, particularly among university students. The transition to university life often involves increased academic and social responsibilities, which frequently lead to a decrease in regular physical activity. This sedentary lifestyle has significant health implications, not only at a physical level but also at a neurocognitive level (1).

Executive functions—a set of high-order cognitive processes including inhibitory control, working memory, and cognitive flexibility—are particularly sensitive to physical activity levels (2). These functions are critical for academic success, self-regulation, and goal-directed behavior. Numerous studies indicate that physical inactivity negatively impacts these cognitive domains. In contrast, regular physical activity has been shown to enhance executive functioning (2).

One key mediator proposed in the relationship between physical activity and brain function is insulin-like growth factor 1 (IGF-1). IGF-1 is a neurotrophic and neuroprotective hormone involved in synaptic plasticity, neurogenesis, and neuronal survival (3). Although not typically used in clinical cognitive assessments, growing evidence suggests that IGF-1 levels correlate with cognitive performance. This is especially evident in domains associated with the prefrontal cortex and hippocampus—regions critical for executive functions. For example, higher peripheral IGF-1 concentrations have been linked to better performance on attention, memory, and processing speed tasks in both aging and young adult populations (4). Moreover, experimental studies show that physical activity induces the expression and release of IGF-1 from peripheral tissues. This circulating IGF-1 can cross the blood–brain barrier and promote brain plasticity (5). This mechanistic link supports the use of IGF-1 as a biological marker of cognitive health and physical activity efficacy, even in young populations where cognitive decline is not clinically apparent (6).

In addition to IGF-1, it is important to recognize that the relationship between physical activity and cognitive health is complex and mediated by multiple, interconnected biological mechanisms. These include the release of brain-derived neurotrophic factor (BDNF), which supports synaptic plasticity and neurogenesis, as well as increased cerebral blood flow, which enhances oxygen and nutrient delivery to key brain regions. Furthermore, exercise-induced structural changes in the hippocampus and prefrontal cortex—areas critically involved in executive functioning—also contribute to cognitive improvements. These mechanisms work synergistically and vary depending on the intensity, duration, and type of physical activity (7). IGF-1 was selected in this study as a relevant and accessible biomarker due to its neurotrophic properties and its responsiveness to exercise. However, it is not the only nor necessarily the most definitive indicator of cognitive outcomes.

High-Intensity Interval Training (HIIT) has emerged as a particularly effective exercise modality for improving these variables. HIIT is characterized by brief periods of high-intensity exercise alternated with recovery periods. It has been shown to improve both executive functions and IGF-1 levels more efficiently than sustained moderate-intensity exercise (4). However, despite its benefits, exercise programs often face the challenge of poor adherence, especially among young populations (5).

To address this barrier, it is crucial to develop exercise programs that are short, flexible, and can be performed at home (4). Combining physical activity with HIIT presents a promising strategy to foster adherence and maximize cognitive and neurophysiological benefits in sedentary young individuals. Increasing the number of daily steps has been consistently associated with improvements in executive functions (8). Regular physical activity, such as walking, induces structural and functional changes in the brain, particularly in areas associated with executive functions like the prefrontal cortex and the hippocampus (9). Light-intensity physical activity stimulates the production of brain-derived neurotrophic factor (BDNF), which promotes neurogenesis and synaptic plasticity. These processes are essential for memory and learning (10). In addition, walking enhances cerebral blood flow. This improves the delivery of oxygen and nutrients to the brain, thereby supporting cognitive health (11). Thus, incorporating walking into daily routines is practical and effective, promoting cognitive health through simple lifestyle changes.

In this context, our research aims to evaluate the chronic effect of high-intensity interval training on executive functions and IGF-1 concentration in young sedentary university students. Based on the preceding findings, it is hypothesized that the intervention groups, especially the HIIT + PA group, will experience a more significant improvement in cognitive functions and IGF-1 levels compared to the control group, due to the combined effects of HIIT and the increase in daily physical activity.

This study is expected to provide evidence on the effectiveness of accessible and flexible HIIT programs, contributing to the formulation of more effective intervention strategies for this population.

## Methods

### Study population

The current study adopts a controlled and randomized experimental design (RCT), employing a sequential approach with three parallel groups and repeated measures assessed at pretest (T1), post-test (T2), and following the conclusion of the follow-up period (T3). All variables were measured at three time points. At the beginning of the training program (T1), after 12 weeks of the HIIT program (T2), and following a 12-week follow-up period (T3) to assess the residual effect.

The study population consisted of healthy and sedentary female university students (22 ± 1.1 years) who did not engage in regular physical activity, defined as less than 150 min of moderate physical activity per week (IPAQ) (12). Sedentary status was determined through a self-reported physical activity questionnaire administered during the initial screening process, where participants confirmed performing less than the recommended 150 min of moderate-intensity activity per week, in line with WHO guidelines.

Inclusion criteria were as follows: (1) age between 18 and 25 years; (2) reflect in the IPAQ questionnaire to be in category 1 or low level of physical activity (12) and therefore not conforming to the minimum recommendations for PA and physical exercise; (3) no diagnosis of neurological, cardiovascular, or metabolic conditions; (4) not taking medications that could affect cognitive function; and (5) commitment to participate in the entire intervention and assessment process. Exclusion criteria included: (1) regular participation in structured exercise programs in the last 6 months; (2) current pregnancy or breastfeeding; and (3) failure to attend at least 80% of the scheduled training sessions; (4) bilingualism. The habitual and indistinct use of two or more languages and (5) playing a musical instrument regularly and continuously, as in the previous criterion, has a positive effect on the subject's cognition.

Sample size estimation was conducted using G*Power 3.1 software. To ensure the validity and generalizability of the results, the sample size was calculated based on the main outcome variable: salivary IGF-1 levels in response to high-intensity physical exercise. The study by Antonelli et al. (13) was used as a reference due to its methodological similarity and comparable sample characteristics. Based on this, a minimum of 25 participants per group was determined to be necessary, assuming a 95% confidence level (*α* = 0.05) and a statistical power of 90%. The sample was increased to 77 participants at baseline to account for potential dropouts.

The initial recruitment aimed to enroll 77 sedentary college women meeting these criteria. After completing the intervention and full assessment process, the final evaluated population consisted of 58 women. Thus, the total number of women included in the final analyses was 20 for the high-intensity interval group (HIIT), 21 for the high-intensity interval group plus physical activity (HIIT + PA), and 17 in the control group (CG). All participants provided written informed consent.

The control group (CG) was instructed to maintain their usual daily routines without engaging in any structured physical activity. Weekly check-ins were conducted to ensure adherence and to confirm no additional interventions were introduced.

Following the 12-week intervention, all groups entered a 12-week follow-up period without structured training or supervision. The purpose was to evaluate whether the effects of the HIIT and HIIT + PA interventions persisted once active supervision ceased.

A detailed account of cohort recruitment and study procedures has been previously documented and published (14), with reference to the ClinicalTrials.gov Identifier: NCT05642169. This study protocol was reviewed and approved by the Ethics Committee for Research Ethics Committee of the Virgen Macarena-Virgen del Rocío University Hospitals (2038-N-20).

### Randomization and blinding

A computer-generated simple randomization sequence was used to assign participants to the HIIT program (HIIT), HIIT plus increased daily physical activity (HIIT + PA) or control group (CG) after the first assessment. Members of the research team involved in the assessments and the data analysis were blinded to the group allocation.

Additionally, the psychologist responsible for administering the cognitive assessments was blinded to the participants’ group assignment to prevent assessment bias. Participants were informed they were involved in a study comparing the effects of different physical activity protocols but were not made aware of the specific hypotheses or which intervention was expected to produce greater cognitive benefits. This partial concealment minimized expectancy effects and performance bias.

A total of 77 women were randomized into the HIIT (*n* = 25), HIIT + PA (*n* = 25) or CG (*n* = 27). Subsequently, participant signed a written informed consent before taking part in the study.

## Procedures

### Exercise groups and increase in physical activity

The training sessions occurred three times a week (Monday, Wednesday, and Friday) over a period of 12 weeks. The intervention groups were divided into 5 groups, each consisting of approximately 10 participants. The training sessions were conducted online, with a coach monitoring the physical actions to ensure proper intensity and technique correction. The exercise program consisted of a 10 min warm-up period with mobility and dynamic stretching exercises, a progressive main part ranging from 24 to 36 min, and a 5-min cool-down with stretching and relaxation. The main part followed a HIIT structure with a density of 1:1 (30 s of exercise and 30 s of rest).

The training programme started at an intensity of 8 on the Borg scale for the first 3 weeks. This first phase served as an adaptation period. Once this first period was completed, 3 weeks were carried out at an intensity of 8.5. This was then increased by 0.5 every 2 weeks until maximum effort was achieved during the last 2 weeks.

The training program followed a progressive overload model: during the first 3 weeks, participants trained at an RPE of 8 (vigorous intensity), which increased progressively toward the final 2 weeks, when participants reached maximum intensity (RPE 9–10).

Full details on the training programme and its progression were published in the following study (14).

In addition to HIIT, the HIIT + PA group was required to achieve a daily goal of at least 10,000 steps. To monitor this, all participants in this group wore wrist-worn physical activity trackers, and the number of daily steps was recorded at the end of each day. Participants submitted these records through a digital platform monitored by the research team.

To support adherence, individualized feedback was provided regularly based on step counts, and specific strategies were implemented to help participants reach their daily goal. These included motivational messages, practical recommendations to increase incidental activity (e.g., walking instead of taking the elevator or public transport), and behavioral prompts during the day.

The number of daily steps was recorded for each participant, and weekly reports were generated. While most participants successfully reached the target on most days, the level of compliance varied slightly across individuals. These deviations were considered in the analysis and reported in the adherence section of the study.

The control group (CG) did not receive any intervention. Participants in this group were instructed to maintain their usual daily routines and refrain from starting any new structured physical activity programs during the study period. They were not provided with guidance or supervision related to physical activity, and their activity was not externally monitored. This group served as a baseline comparison to evaluate the effects of the HIIT and HIIT + PA interventions.

### Measured outcomes

All the variables listed below were recorded at 3 moments (T1, T2 and T3).

#### Body composition variables

Body composition variables (fat mass and muscle mass) were estimated using segmental bioelectrical impedance analysis (BIA) with a Tanita BC-418 device (Tokyo, Japan), a valid and reliable non-invasive method (15). Measurements were taken under standardized conditions, including at least 4 h of fasting, avoidance of intense exercise and caffeine, and urination 30 min before the test (Table 1).

**Table 1:** Anthropometric characteristics of the participants at baseline (Mean ± SD).

Variable	HIIT(*n* = 20)	HIIT + PA (*n* = 21)	CG (*n* = 17)
Weight (Kg)	61.95 ± 15.3	58.59 ± 9.6	62.52 ± 8.6
Height (cm)	163.9 ± 4.6	161.2 ± 5.8	161.1 ± 5.2
Fat mass (%)	30.22 ± 8.9	30.27 ± 6.4	31.78 ± 5.5
Muscle mass (Kg)	40.1 ± 4.2	38.3 ± 3.2	40.1 ± 3.5

HIIT, high interval intensity training group; HIIT + PA, high interval intensity training group plus increase of physical activity; Mean ± standard deviation; CG, control group.

#### Biochemical determinants

Saliva samples were collected under standardized conditions at three time points: baseline (T1), post-intervention (T2), and post-follow-up (T3). All samples were collected early in the morning (between 9:00 and 10:00 a.m.) after an overnight fast of at least 8 h. This protocol was applied uniformly to all participants to control for circadian and dietary influences on salivary biomarkers. Saliva samples were collected under fasting conditions in the early morning, using the passive drool method, which has been shown to provide reliable hormone quantification (16). After collection, the samples were centrifuged at 1,500 g for 15 min and stored at −80°C. IGF-1 levels were determined using enzyme-linked immunoassays (ELISA) according to the manufacturer's protocol (Elabscience, USA), and all samples were measured in duplicate.

The decision to use salivary IGF-1 instead of serum was based on considerations of feasibility and non-invasiveness, particularly given the study population of young, healthy individuals. Salivary collection methods reduce participant burden, increase compliance, and allow for repeated measures without the need for venipuncture. Furthermore, recent studies have demonstrated a significant correlation between salivary and serum IGF-1 levels, supporting its validity as a biomarker in exercise-related interventions (17, 18). Thus, salivary IGF-1 represents a practical and sufficiently sensitive alternative for assessing endocrine responses to physical activity in field-based and non-clinical research settings.

Saliva was collected in a fasting state using the passive drooling method, then centrifuged and stored at −80°C. Enzyme-linked immunoassays (ELISAs) were used to measure IGF-1 concentration in duplicate. Samples were added to microplate wells with specific antibodies and incubated with Avidin conjugated with Horseradish Peroxidase (HRP). Only wells containing IGF-1 showed a color change when 3,3′, 5,5′-Tetramethylbenzidine (TMB) substrate solution was added. Colorimetric changes were induced by TMB substrate in the presence of IGF-1 and quantified using a standard curve. Concentration was determined by comparing with a standard curve using Elabscience kits (Elabscience Biotechnology Inc.)

Although salivary IGF-1 represents the unbound (free) fraction of circulating IGF-1, previous research indicates that it correlates meaningfully with systemic levels, particularly in response to acute physical activity (17). This supports its use as a non-invasive and reliable biomarker in young, healthy individuals undergoing physical training.

#### Executive functions

The measurements of executive functions were carried out with the collaboration of a psychologist specialized in neurocognitive testing. Three standardized test tests were used:

The Stroop Test (ST) was employed to evaluate inhibitory control, utilizing a digitized version (19). This brief assessment measures interference during task performance. Variables such as time and the number of intrusion errors resulting from inhibitory control failures were assessed.

The Wisconsin Card Sorting Test (WCST) primarily assesses cognitive flexibility (20). This test involves 64 cards with various combinations. Measured variables include the number of correct responses, identified categories, perseverative responses, set maintenance failures, among others. The WCST has demonstrated high reliability and validity (21).

Finally, the Digit Span Test (DST) measures working memory. It consists of two measures: forward and backward. The number of digits reached without errors, both in forward and backward order, is evaluated (22). The outcomes are summarized in Table 2 along with other dependent variables.

**Table 2:** Summary of variables related to executive function.

Stroop test (ST)	Nomenclature
Interference time	ST-I-T
Interference correct responses	ST-I
Wisconsin card test (WCST)	
Correct categories	WCST-C
Correct responses	WCST-R
Perseverative error	WCST-*P*-E
Digit span test (DST)	
Forward memory	DST-F
Backward memory	DST-B
Total correct responses (forward + backward)	DST-T

All assessments followed standardized administration procedures to ensure reliability, and references have been added to support the validity of the tests used. Participants were scheduled at the Exercise Laboratory of the University of Seville. After following the standardized protocol the previous day, they first underwent height and body composition assessments. This was followed by 5 min of seated rest to ensure a resting state before saliva collection using the passive drool method (5 ml into sterile polypropylene tubes). Finally, executive functions were assessed using the ST, WCST, and DST.

### Statistical analysis

Once the data were obtained from the three research groups (HIIT, HIIT + PA, and CG), they were compared at the beginning, after 12 weeks of intervention, and 12 weeks after the end of the training program. Only women who attended at least 80% of the exercise sessions were included in the statistical analyses. To assess intergroup differences in parametric variables, a one-way ANOVA with three levels was conducted. If significant differences in means between groups were observed, the *post hoc* Bonferroni test was employed to identify the specific group(s) showing differences. In cases of unequal variances, the T3 Dunnett test was utilized. For variables that did not meet the assumption of normality, the non-parametric Kruskal–Wallis test was performed. If significant differences were detected, the Mann–Whitney test was applied to evaluate differences between groups.

Within-group comparison was conducted to contrast differences between groups after the intervention. For variables that met normality, repeated measures ANOVA was applied, and for those that did not, Friedman and Wilcoxon signed-rank tests were used. Cohen's criteria (23) were followed for effect size analysis. Prior to the main analysis, the dataset was screened for outliers using a ± 3 standard deviation criterion. In the Digit Span Backward (DST-B) variable, three extreme values were identified and excluded from the analysis All data were analyzed using SPSS v.26 (IBM Corp., Armonk, NY, USA), with a confidence interval of 95%, and a significance level of *p* ≤ 0.05.

## Results

**Figure 1:**
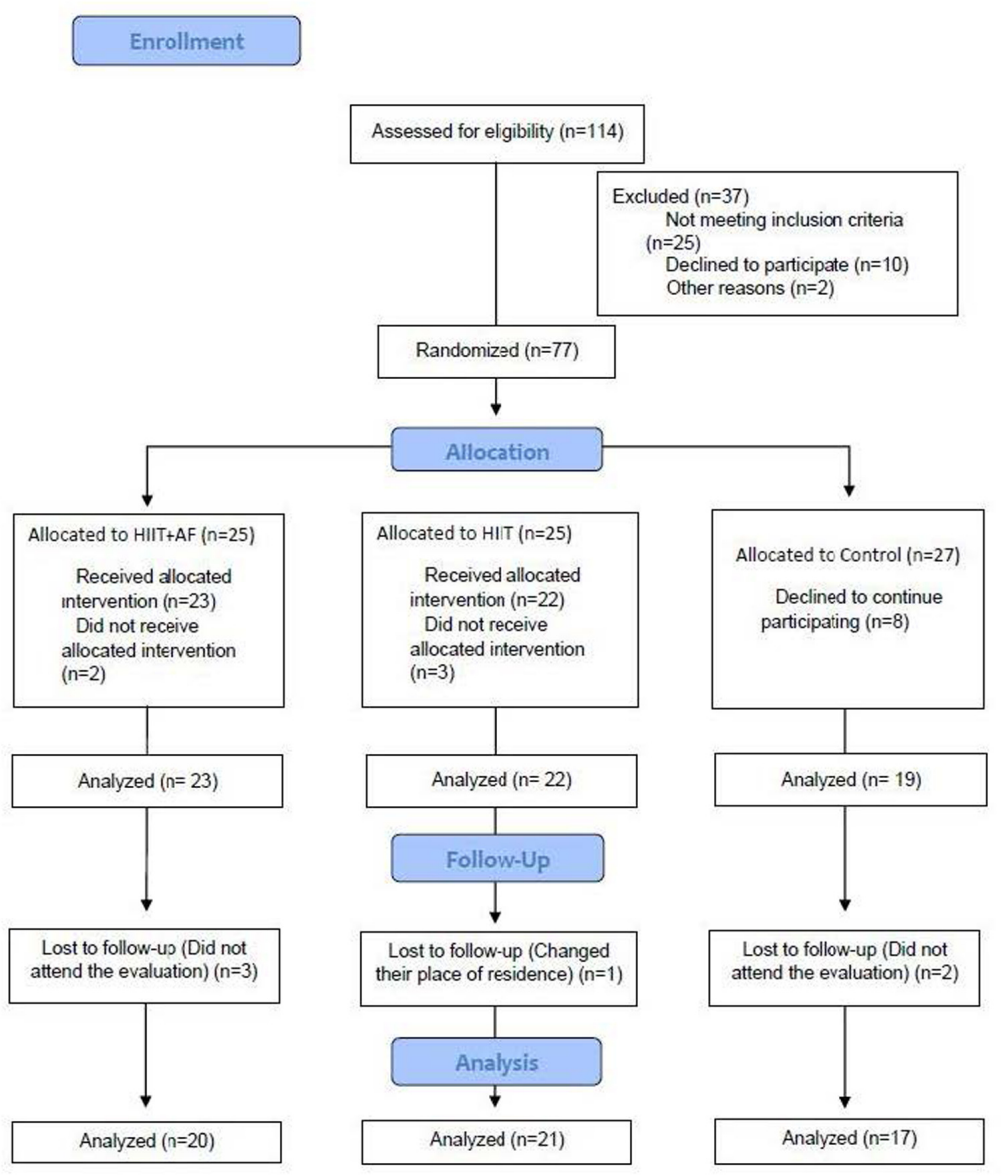
Flowchart represents different stages in the research process*.*

Once the sample was selected, it consisted of a total of 77 subjects (HIIT + PA, *n* = 25; HIIT, *n* = 25; GC, *n* = 27). After completing the entire training period and follow-up, due to dropouts at some point in the research, the final analyzed sample was of 58 women (HIIT + PA, *n* = 20; HIIT, *n* = 21; GC, *n* = 17) (Figure 1).

Focusing on the main study variable, the initial concentration of the 3 groups showed no significant between-groups differences (*p* = 0.336). Regarding executive functions (ST, WCST, and DST), no differences between groups were found in each executive function at the baseline (*p* > 0.05). Only DST-B showed differences between groups at baseline (*p* = 0.02).

For salivary IGF-1 concentration, the repeated measures ANOVA did not show significant effects for time (*p* = 0.501), group (*p* = 0.677), or group × time interaction (*p* = 0.404), suggesting no robust differences across conditions or over time at the group level. Despite this, descriptive data show numerical increases in IGF-1 levels in the HIIT and HIIT + PA groups between T1 and T3 (e.g., HIIT mean from 1.1 to 3.4 ng/ml; HIIT + PA from 1.1 to 3.4 ng/ml), whereas the control group remained relatively stable (from 1.7 to 2.3 ng/ml).

These increases, however, should be interpreted with caution given the high variability in the T3 data (standard deviation = 4.6 in both intervention groups) and the non-significant overall effects. As such, the pairwise or within-group comparisons are presented for descriptive purposes only and should not be interpreted as confirmatory evidence of effectiveness.

Although no significant group or interaction effects were found in the repeated measures ANOVA for IGF-1 concentration (group *p* = 0.677; interaction *p* = 0.404), significant changes over time were observed in the within-group analyses (Table 3) were observed in both the HIIT and HIIT + PA groups (HIIT: *p* = 0.002, d = 0.96, Δ = 29.6; HIIT + PA: *p* ≦ 0.001, d = 0.78, Δ = 28.8). When observing the residual effect 3 months after the conclusion of the training program, only the HIIT (*p* = 0.036, d = 0.42, Δ 50.5%) and HIIT + PA groups (*p* = 0.03, d = 0.48, Δ 77.3%) demonstrated significant changes in IGF-1 concentration compared to the CG.

**Table 3:** IGF-1 salivary concentration at the three evaluation timepoints T1, T2, and T3 (*n* = 58).

Variable	Group	T1 Media (sd)		T2 Media (sd)		T3 Media (sd)		P valor time	*η*^2^ partial	P valor group	η^2^ partial	P valor group*time	η^2^ partial
IGF-1 (ng/ml)	HIIT	1.1	(0.3)	1.5	(0.3)	2.4	(2.4)	0.001*		0.845		0.817	
	HIIT + PA	1.2	(0.1)	1.5	(0.5)	2.5	(2.0)		0.23		0.01		0.01
	CG	1.1	(0.1)	1.6	(0.5)	2.0	(1.3)						

Ng, nanogram; SD, standard deviation; ml, milliliter.

* Significant differences (*P* < 0.05).

In terms of inhibitory control (ST) (Table 4), a significant effect of time was observed in the ST-I-T (interference time) variable, reflecting an overall reduction in completion time across the sample from T1 to T2 and T3 (*p* < 0.001). However, no significant group (*p* = 0.732) or grou*p* × time interaction effects (*p* = 0.563) were found, suggesting that improvements were not specific to any intervention group.

**Table 4:** Differences between different phases and groups in TS at time T1, T2 and T3.

Variable	Group	T1 Media (sd)		T2 Media (sd)		T3 Media (sd)		*p* valor time	η^2^ partial	*p* valor group	η^2^ partial	*p* valor group*time	η^2^ partial
ST- I -T (s)	HIIT	37.5^a^^,^^b^	(16.0)	29.8	(7.45)	29.7	(7.6)	<.001*		.387		.542	
	HIIT + PA	33.5^a^	(11.1)	28.4	(10.1)	28.9	(11.6)		.208		.034		.027
	CG	37.9^b^	(12.0)	34.5	(11.3)	31.2	(8.3)						
ST- I (N° correct responses)	HIIT	28.1^b^	(4.5)	27.0	(2.8)	26.2	(1.8)	.520		.280		.138	
	HIIT + PA	26.8	(3.6)	27.5	(3.0)	27	(3.5)		.012		.045		.061
	CG	26.1	(2.9)	25.6	(1.6)	26.2	(1.6)						

S, seconds; SD, standard deviation; ST, stroop test; ST-I-T, total time; ST-I, number of correct responses.

* Significant differences (*P* < 0.05).

^a^ Significant differences between T1 and T2.

^b^ Significant differences between T1 and T3.

Descriptively, both the HIIT and HIIT + PA groups exhibited reductions in interference time—HIIT from 37.5 s to 29.8 s, and HIIT + PA from 33.5 s to 28.4 s—while the control group also improved from 37.9 s to 34.5 s. These reductions persisted at follow-up (T3).

For the number of correct responses (ST-I), only the HIIT + PA group showed a significant effect over time (*p* = 0.003). However, group (*p* = 0.116) and interaction (*p* = 0.126) effects were not significant, indicating that the improvement in accuracy is likely not attributable to the intervention *per se*.

It is important to highlight that while these statistical improvements are significant, their practical impact on daily functioning, academic performance, or other real-world activities remains uncertain. Future studies could explore whether such changes in inhibitory control translate into meaningful cognitive benefits outside of the laboratory setting.

Regarding cognitive flexibility, significant time effects were observed in both WCST-C (*p* = 0.013) and WCST-R (*p* = 0.001), indicating improvements over time in the number of completed categories and correct responses. However, no significant group or interaction effects were found for either variable (all *p* > 0.6), suggesting that these improvements were not specific to the intervention groups.

For WCST-E-P (Perseverative Errors), a significant group effect was found (*p* = 0.028), indicating overall differences between groups, but no significant time or group × time interaction effects were observed. This suggests that although groups differed in the number of perseverative errors, these differences were not clearly driven by the training program.

The improvements in cognitive flexibility, although statistically significant, suggest potential benefits in executive functioning, which might influence tasks requiring adaptability, such as problem-solving or decision-making, which are essential for academic and professional settings

In the working memory domain (Table 6), the DST-B (Digit Span Backward) revealed a significant main effect of time (*p* < 0.001, *η*^2^ = 0.953), indicating improvements across all groups. However, neither the group effect (*p* = 0.843) nor the grou*p* × time interaction (*p* = 0.286) were statistically significant, suggesting the changes were not specific to any intervention.

For the DST-F (Forward), no significant time effect was observed (*p* = 0.565), nor were there significant differences between groups (*p* = 0.320) or interaction effects (*p* = 0.531).

The DST-T (Total) showed a significant time effect (*p* = 0.001), but similar to the other measures, the group × time interaction was not significant (*p* = 0.081). Improvements in the HIIT group between T1 and T2 (Δ = 1.3 points) were numerically notable, but cannot be attributed to the intervention with statistical certainty.

## Discussion

The purpose of the present study was to test the hypothesis that a 12-week HIIT program implemented with an increase in daily PA (HIIT + PA) enhances, to a greater extent, both IGF-1 concentration and executive functions in sedentary young women than a similar program (HIIT) but not implemented.

Furthermore, after 12 weeks following the completion of the training, these same variables measured would remain above that observed at baseline.

The results show how a 12-week HIIT training program can significantly improve IGF-1 concentration in sedentary young college women. In addition, it was observed that the improvements were increased to a greater extent after the follow-up evaluation, with the HIIT + PA group obtaining a higher percentage of change.

On the other hand, with respect to executive functions, only the HIIT + PA group obtained greater efficiency in inhibitory control (ST) after the training program. Regarding cognitive flexibility (WCST), there are certain improvements in the HIIT and HIIT + PA groups after the HIIT program, but it is after the Follow-up when the HIIT + PA group shows a higher performance. Regarding working memory, the HIIT group obtained a significant change after the training program and after the follow-up period in the DST backward test (DST-B), which requires a greater demand on working memory.

### Changes in IGF-1

Our results did not show statistically significant changes in IGF-1 concentrations over time or between groups. Although descriptive increases were observed in the HIIT and HIIT + PA groups at the three-month follow-up (T3), these changes were not statistically significant in the group, time, or interaction effects (*p* > 0.40). The response of IGF-1 to exercise is not uniformly consistent, as evidenced by contradictory findings in the literature (24). Studies with young adults suggest that exercise intensity is a key factor in growth hormone release, including IGF-1, with effects increasing in a dose-response manner (25), which can influence brain structure and function (26). Given our study's focus on HIIT, intensity likely played a central role in the observed tendencies.

Nevertheless, other studies have reported no significant changes or even reductions in IGF-1 levels following similar interventions (27). This variability highlights the complex interplay of factors such as training duration, baseline fitness, and individual response. Still, the evidence suggests that high-intensity exercise triggers metabolic and mechanical stress, which activates key cellular pathways such as the Akt/mTOR cascade—critical for protein synthesis and IGF-1 production (28). The IGF-1/IGF1-BP axis modulates the availability and half-life of IGF-1, affecting cellular proliferation and neuroplasticity (29).

Regarding the residual effect at T3, although IGF-1 concentrations appeared numerically elevated in the HIIT and HIIT + PA groups compared to baseline, the statistical analysis did not confirm significant group differences or interaction effects. These tendencies, while potentially indicative of long-term physiological adaptations, must be interpreted cautiously. Effect sizes and within-group changes can suggest patterns, but without significant omnibus tests, *post hoc* interpretations are limited and should not be overstated.

This aligns with previous literature showing mixed results on IGF-1 responses depending on protocol specifics and individual variability (30–33). While IGF-1 has been widely studied in relation to brain health and neuroplasticity, it is not a clinically validated biomarker of executive function. Therefore, while increases observed in some participants may suggest neurobiological adaptations, the current results do not provide robust statistical evidence to support this as a generalizable effect**.**

### Changes in executive functions

Recent meta-analyses have investigated the effects of physical exercise on executive functions in young adults (30) showing overall modest effects. While working memory tends to benefit more robustly, inhibitory control shows smaller or null effects (Hedges' g = 0.16). These mixed results suggest that the effect of exercise may depend on the specific cognitive domain, intervention characteristics, and participant profile.

In our study, although reductions in interference time (ST-I-T) were observed, these improvements were not specific to the intervention groups, as no group or interaction effects were found. Therefore, the observed improvements may reflect general learning effects, repeated testing, or other non-specific factors, rather than a direct result of the training programs.

In contrast, only the HIIT + PA group showed a significant improvement over time in the number of correct responses, suggesting a possible selective benefit in response accuracy. Nevertheless, the absence of significant between-group differences tempers this conclusion, and prevents us from confidently attributing this change to the training intervention.

This aligns with prior findings indicating that changes in inhibitory control may require longer interventions or be influenced by individual variability, particularly in sedentary women (34, 35). The prefrontal cortex, central to inhibitory control, may not respond immediately to exercise stimuli and may benefit more from sustained engagement or cognitively demanding exercise modalities (33).

Our results show statistically significant improvements over time in the WCST-C and WCST-R variables (Table 5), indicating enhanced cognitive flexibility across the sample. However, since no group × time interaction was found, these improvements cannot be conclusively attributed to the intervention. They may reflect general cognitive engagement, learning effects, or other nonspecific influences over the course of the study.

**Table 5:** Differences between different phases and groups in WCST at time T1, T2 and T3.

Variable	Group	T1 Media (sd)		T2 Media (sd)		T3 Media (sd)		*p* valor time	η^2^ partial	*p* valor group	η^2^ partial	*p* valor group*time	η^2^ partial
WCST-C	HIIT	3.9	(0.2)	4.2	(0.3)	4.3	(0.2)	.013*	.69	.619	.619	.719	.69
	HIIT + PA	3.8	(0.2)	4.1	(0.2)	4.6^b^	(0.2)						
	GC	3.8	(0.2)	4.1	(0.3)	4.1	(0.2)						
WCST-R	HIIT	49.3	(3.6)	52.5^a^	(3.6)	53.3^b^	(2.8)	.001*	.201	.590	.019	.497	.03
	HIIT + PA	50.9	(3.7)	53.1^a^	(3.2)	54.5^b^	(3.1)						
	GC	51.9	(3.3)	53.7	(3.0)	53.8	(3.8)						
WCST-E-P	HIIT	6.8	(2.8)	7.1^c^	(1.0)	6.3	(0.8)	.097	.097	.028*	.122	.223	.223
	HIIT + PA	6.3	(2.5)	5.7	(1.9)	5.8	(1.2)						
	GC	7.9^a^	(2.4)	5.7	(1.7)	6.8	(0.8)						

S, seconds; SD, standard deviation; WCST, Wisconsin card sorting test; WCST-C, completed categories; WCST-R, correct responses; WCST-P-E, perseverative errors.

* Significant differences (*p* < 0.05).

^a^ Significant differences between T1 and T2.

^b^ Significant differences between T1 and T3.

^c^ Significant differences between T2 and T3.

**Table 6:** Differences between different phases and groups in DST at time T1, T2 and T3.

Variable	Group	T1 Media (sd)		T2 Media (sd)		T3 Media (sd)		*p* valor time	η^2^ partial	*p* valor group	η^2^ partial	p valor group*time	η^2^ partial
DST-F	HIIT	5.8	(1.3)	6.25	(0.8)	6.2	(1.1)	.565		.320		.531	
	HIIT + PA	6.1	(1.3)	6.33	(0.9)	6.3	(1.2)		.01		.041		.028
	GC	5.9	(0.8)	5.71	(1.1)	5.8	(1.2)						
	HIIT	4.5	(1.2)	5.4^a^	(1.3)	5.2^b^	(1.1)						
DST-B	HIIT + PA	5.4	(1.2)	5.52	(1.4)	5.9	(3.1)	<.001*	.127	.041*	.109	.128	.062
	GC	4.5	(1.6)	4.53	(1.1)	5.2^b^	(3.8)						
DST-T	HIIT	10.3	(2.0)	11.6^a^	(1.5)	11.5^b^	(1.8)	.001*		.073		.081	
	HIIT + PA	11.6	(1.9)	11.8	(2.0)	12.3	(2.1)		.113		.091		.072
	GC	10.5	(2.0)	10.2	(2.0)	11.0	(2.2)						

DST, digit span test; DST-F, forward; DST-B, backward; DST-T, total (forward + backward); s, seconds; SD, standard deviation.

* Significant differences (*P* < 0.05).

^a^ Significant differences between T1 and T2.

^b^ Significant differences between T1 and T3.

In WCST-R, although both HIIT and HIIT + PA groups showed increased scores from baseline to T3, the lack of a significant interaction effect (*p* = 0.719) suggests that these gains were not exclusive to the intervention groups, and may have occurred regardless of training.

The only significant between-group effect was observed in WCST-E-P (*p* = 0.028), indicating group-level differences in cognitive rigidity, but not necessarily as a result of the intervention, since neither time nor interaction effects were significant. Interestingly, the control group showed some reduction in perseverative errors over time, which may be influenced by repeated testing rather than specific training-related cognitive adaptation.

These findings differ from previous reports suggesting that structured physical training, particularly HIIT, may influence cognitive flexibility. Meta-analyses such as those by Haverkamp et al. (30) and Hsieh et al. (35) report small or nonsignificant changes in flexibility-related outcomes, supporting our interpretation that these effects may be subtle, delayed, or require complementary interventions such as broader lifestyle changes or cognitive engagement.

Working memory is often one of the most sensitive executive functions to benefit from physical activity in young adults (30, 34). In the present study, the most pronounced improvements were found in the DST-B, with a highly significant time effect (*p* < 0.001, *η*^2^ = 0.953), suggesting enhanced capacity for manipulating and recalling information. Nevertheless, the lack of significant group or interaction effects indicates that these improvements were not exclusive to the intervention groups.

In contrast, DST-F did not show significant changes over time (*p* = 0.565), and DST-T, although showing a significant time effect (*p* = 0.001), also lacked significant group-specific effects (*p* = 0.081 for group × time interaction). These findings suggest that all groups, including the control, may have benefited from repeated exposure to the test or from external factors unrelated to the intervention, such as academic engagement or increased mental stimulation during the study period.

Our results partially align with previous literature, where HIIT has shown mixed effects on working memory. While some studies have reported improvements (34), others, like (4)**,** found no significant changes in working memory after similar HIIT protocols in sedentary young women. These inconsistencies may be due to individual differences, intervention duration, cognitive task sensitivity, or external lifestyle factors.

Taken together, although statistically significant changes over time were observed—especially in DST-B—they cannot be confidently attributed to the HIIT or HIIT + PA interventions. Future studies should explore longer intervention periods, more ecologically valid cognitive tasks, and control for confounding lifestyle factors to clarify the relationship between exercise and working memory improvements.

Additionally, although the control group did not receive any intervention, an unexpected increase in IGF-1 levels was observed at T3. This finding, while not aligned with our initial hypothesis, may reflect individual variability or external lifestyle factors not controlled during the study. Future research should further investigate potential causes of such hormonal fluctuations in inactive populations.

Future studies that can administer a measurement of structural change via MRI could generate more information and help us to understand the role of HIIT in all the changes produced, both at the biological, functional and structural level in this population.

We also recognize that many configurations can be found to develop a HIIT training programme, which may have a different effect to those found. However, it is important to note that the study was not designed to uncover such information; instead, it was designed to complement the current literature by demonstrating that HIIT provides improvements in IGF-1 concentration and executive performance, and furthermore, if supplemented with increased daily physical activity, the benefits may be greater.

These findings carry important translational implications. The observed improvements in executive functions, particularly inhibitory control and cognitive flexibility, suggest that HIIT—especially when combined with an increase in daily physical activity—may serve as an effective and time-efficient intervention to support cognitive well-being in sedentary young adults. Given the growing interest in lifestyle-based approaches to mental and cognitive health, these results could help inform physical activity guidelines that incorporate high-intensity interval formats as part of broader strategies aimed at enhancing executive functioning. Further studies are needed to explore how such interventions can be adapted and implemented at the population level.

## Conclusions

In summary, this study provides preliminary evidence that a 12-week HIIT program, particularly when combined with an increase in daily physical activity (HIIT + PA), may contribute to improvements in executive functions—most notably in inhibitory control and cognitive flexibility—in sedentary young women. Although IGF-1 levels and working memory also showed favorable changes over time, these effects could not be definitively attributed to the intervention due to the lack of significant interaction effects. The improvements observed across all groups, including the control group, suggest potential contributions from repeated testing, academic demands, or unmeasured lifestyle factors.

While the overall impact of the interventions was modest, the HIIT + PA condition consistently showed more favorable trends, supporting the notion that combining structured exercise with broader activity-based behavioral changes may enhance cognitive outcomes. These findings underscore the need for future research incorporating longer intervention periods, objective neuroimaging assessments, and tighter control of external variables to better understand the mechanisms underlying exercise-induced cognitive changes. Ultimately, HIIT—when integrated into a physically active lifestyle—emerges as a promising, time-efficient strategy to support cognitive health in young sedentary populations.

## Data Availability

The raw data supporting the conclusions of this article will be made available by the authors, without undue reservation.
